# The Role of Extracellular Vesicles in Cutaneous Remodeling and Hair Follicle Dynamics

**DOI:** 10.3390/ijms20112758

**Published:** 2019-06-05

**Authors:** Elisa Carrasco, Gonzalo Soto-Heredero, María Mittelbrunn

**Affiliations:** 1 Immunometabolism and Inflammation Laboratory, Department of Cell Biology and Immunology, Centro de Biología Molecular Severo Ochoa (CBMSO) Consejo Superior de Investigaciones Científicas-Universidad Autónoma, 28049 Madrid, Spain; gonzalo.soto@cbm.csic.es (G.S.-H.); mmittelbrunn.imas12@h12o.es (M.M.); 2 Department of Molecular Biology, Faculty of Sciences, Universidad Autónoma de Madrid (UAM), 28049 Madrid, Spain; 3 Regulation of Cellular Homeostasis Laboratory, Area of Rare and Genetically Based Diseases, Instituto de Investigación Hospital 12 de Octubre, 28041 Madrid, Spain

**Keywords:** extracellular vesicles, exosomes, apoptotic bodies, skin, hair follicles, hair cycle, stem cells, immune cells

## Abstract

Extracellular vesicles (EVs), including exosomes, microvesicles, and apoptotic bodies, are cell-derived membranous structures that were originally catalogued as a way of releasing cellular waste products. Since the discovery of their function in intercellular communication as carriers of proteins, lipids, and DNA and RNA molecules, numerous therapeutic approaches have focused on the use of EVs, in part because of their minimized risk compared to cell-based therapies. The skin is the organ with the largest surface in the body. Besides the importance of its body barrier function, much attention has been paid to the skin in regenerative medicine because of its cosmetic aspect, which is closely related to disorders affecting pigmentation and the presence or absence of hair follicles. The use of exosomes in therapeutic approaches for cutaneous wound healing has been reported and is briefly reviewed here. However, less attention has been paid to emerging interest in the potential capacity of EVs as modulators of hair follicle dynamics. Hair follicles are skin appendices that mainly comprise an epidermal and a mesenchymal component, with the former including a major reservoir of epithelial stem cells but also melanocytes and other cell types. Hair follicles continuously cycle, undergoing consecutive phases of resting, growing, and regression. Many biomolecules carried by EVs have been involved in the control of the hair follicle cycle and stem cell function. Thus, investigating the role of either naturally produced or therapeutically delivered EVs as signaling vehicles potentially involved in skin homeostasis and hair cycling may be an important step in the attempt to design future strategies towards the efficient treatment of several skin disorders.

## 1. Introduction

**Figure 1:**
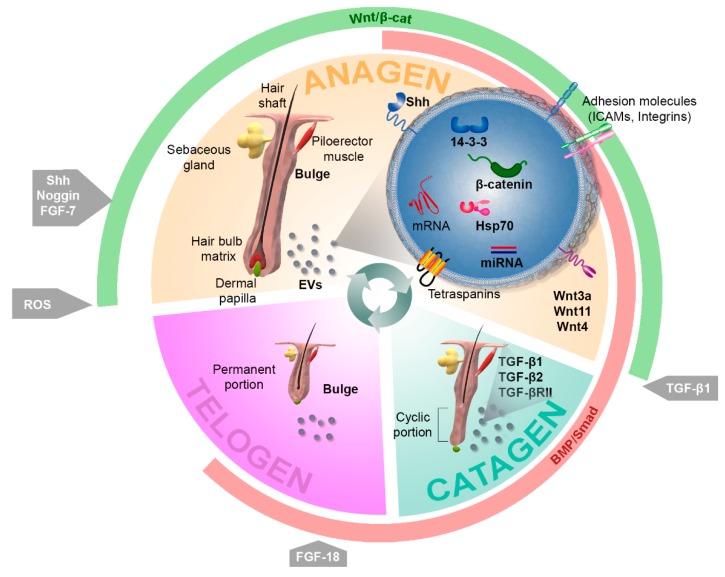
Regulation of the hair follicle cycle. Hair follicles continuously cycle, undergoing consecutive phases of growing (anagen), regression (catagen), and resting (telogen). The fine regulation of hair follicle dynamics globally depends on the coordinated alternation between Wnt/β-catenin (green) and BMP/Smad (red) signals, which mainly emanate from the dermal papilla and control the behavior of follicular epithelial cells. Several factors and molecules have been found to affect different steps of the cycle, including Sonic hedgehog (Shh), Noggin, FGF-7, and reactive oxygen species (ROS), which work as inductors of growth; TGFβ-1, which is involved in catagen onset; and FGF-18, which regulates telogen. The role of extracellular vesicles (EVs) in skin has been assessed to date with a major focus on their effects on the wound healing process but suggesting substantial effects on hair cycling. For instance, EVs containing Wnt3a, Wnt11, Wnt4, β-catenin, and 14-3-3 proteins may contribute to hair growth by enhancing Wnt signaling; HSP70 containing exosomes have been related to pigmentation defects; and the routes involving Pi3K/AKT, MAPK/ERK, STAT3, and IGF1 have been connected with exosome-mediated effects in skin, therefore becoming potential targets for EV-mediated therapeutic approaches.

The skin is the largest organ in the body in terms of extension (1.5–2 m^2^ in humans) and comprises around 16% of total bodyweight [1]. As the major body barrier, it plays an essential role in both stimuli sensing and defense against environmental insults and pathogens. The outer skin layer is the epidermis, a stratified epithelium of ectodermal origin with a high turnover rate. It is mainly built by keratinocytes but also contains other cell types, such as melanocytes, Merkel cells, and Langerhans cells, which are involved in pigmentation, sensing, and immune response, respectively. Resting below the epidermal basal membrane is the dermis, a mesenchymal layer of connective tissue containing collagen fibers and fibroblasts as well as blood vessels and nerve endings. Finally, the composition of the subjacent hypodermal tissue is dependent on the part of the body (e.g., fat, connective tissue, bone). Skin appendices include hair follicles (HFs) and glands. HFs are important for warmth function in mammals but also account for social and cosmetic features in humans. The HF cycle continuously occurs over the life of the organism, involving successive phases of rest (telogen), growth (anagen), and regression (catagen) [2] (Figure 1). Directly below the sebaceous gland and at the level of insertion of the piloerector muscle, the HF bulge, which hosts a major reservoir of skin stem cells (SCs), is located [3]. Due to ease of accessibility and knowledge collected about the location and function of skin SCs, the skin in general, and the HF in particular, have drawn much attention as targets for regenerative treatments. Among these, the use of extracellular vesicles as a safer alternative in comparison with cell-based approaches is gaining importance.

Extracellular vesicles (EVs) comprise a heterogeneous variety of membrane-enclosed structures in terms of size, mechanisms of biogenesis, composition, cargo, and functions. They contain transmembrane proteins and enclose components derived from the donor cell that may include proteins, lipids, and DNA and RNA molecules [4,5,6]. Most types of cells can secrete EVs, from bacterial to animal—including human—to plant cells, which suggests that such an evolutionary conserved mechanism [7] could be playing an essential role in cellular function. EVs can be isolated from body fluids or cell culture supernatants and are nowadays becoming central signaling vehicles and critical players in cell-cell communication, thus being implicated in physiological and pathological processes [8].

According to their route of biogenesis, EVs are generally classified into three subtypes, namely, exosomes, microvesicles, and apoptotic bodies, with the former having been more frequently connected to skin regenerative processes. Exosomes are 30–150 nm vesicles which originate via the endocytic pathway. Normally, inward budding of the plasma membrane or fusion of vesicles gives rise to early endosomes, which in turn can undergo inward budding, forming so-called multivesicular bodies (MVBs). The intraluminal vesicles created by this process can either be directed to degradation in late endosomes, involving lysosomes, or can be secreted to the extracellular space by exocytosis, which is mediated by the fusion of MVBs with the plasma membrane [9,10]. Since the first characterization of exosomes in the 1980s [11,12] and in light of later studies proving their role beyond the clearance of waste products as immune players and even as anti-tumor vaccines [13,14], much research has been directed at understanding their mechanisms of biogenesis. It is well established that Rab GTPase family members play a key role in the modulation of exosome secretion, mainly along the pathway that involves Endosomal Sorting Complexes Required for Transport (ESCRT), though the process can also occur in an ESCRT-independent manner, in which tretraspanins (CD81, CD9, and CD63) and lipids play a crucial role [7,10]. Microvesicles are larger lipid bilayer membrane-enclosed structures (0.1–1 µm) which originate via outward budding of the cell membrane. Although their mechanism of formation has not yet been uncovered in detail, it is known to involve a rearrangement of plasma membrane phospoholipids, enzymatic processes, and cytoskeletal-mediated contraction, which eventually allows for the fission of the membrane protrusions. Finally, relatively large (up to several microns) apoptotic bodies are formed by outward blebbing of the plasma membrane of apoptotic cells, meaning they usually contain cellular fragments [15]. Despite the increasing consensus about EV classification, it is hard to distinguish them once they reach the extracellular space. Their interaction with recipient cells can occur in different ways: ligand-receptor interaction; internalization by clathrin-dependent endocytosis, caveolin-mediated uptake, macropinocytosis, or phagocytosis mediated by specific receptors; and direct fusion with the plasmatic membrane of the recipient cell, thereby involving the release of EV content in the cytoplasm of the recipient cell [16]. These processes can affect a number of key cellular signaling pathways that modulate essential cellular processes, such as proliferation, differentiation, migration, and cell death.

## 2. Extracellular Vesicles in Cutaneous Regenerative Medicine

### 2.1. Use of Extracellular Vesicles to Boost Skin Regeneration

The skin wound healing process comprises four stages: (1) hemostatic, (2) inflammatory, (3) proliferative, and (4) remodeling [17,18]. During this process, the skin is sequentially: (1) activated to recruit repairing cell types; (2) cleaned of pathogens by the immune system; (3) stimulated to provoke the proliferation of fibroblasts and the production of the extracellular matrix; and (4) closed by the structural adjustment of the newly produced extracellular matrix. EVs obtained from a plethora of cell types, mainly including mesenchymal stem cells (MSCs) of different origin, but also dermal papilla (DP) cells, amniotic epithelial cells, keratinocytes, and endothelial progenitors, among others, have been tested within different models of skin injury, such as skin wounds in healthy and diabetic rodents or severe burns in rats (as reviewed in [17,19,20]). Key steps of the healing process have been proven to benefit from the action of EVs, including cell proliferation, migration [21,22,23,24,25], angiogenesis [22,26,27,28,29] and collagen deposition [22,25,28,29,30], and are mainly mediated by enhanced AKT/ERK and Wnt signaling. Overall, the effects of EVs can be summarized in accelerated wound healing and reduced scar formation.

Additionally, it has been suggested that multipotent neural SCs obtained from HFs can serve as exosome producers, based on the beneficial effects of a conditioned medium for the treatment of ischemia-reperfusion-induced lung injury in a rat model [31]. Hence, HFs can also be postulated as a source of exosome-producing SCs.

However, robust conclusions and defined clinical protocols are still hard to define because of the lack of consensus regarding the source of EVs used in different studies, the models chosen to test EV treatments, and the methods used for EV delivery. Additionally, the technical procedures used to isolate, purify, and quantify exosomes and microvesicles, which have been fairly extensively reviewed [32], are indeed a crucial point to consider in order to allow data standardization. For these reasons, the molecular mechanisms of the action of EVs in wound healing need to be further characterized, with special attention paid to simultaneous effects on hair growth.

### 2.2. Regulation of the Pigmentation Process by Exosomes

The cell population of melanocytes, which are resident in the skin but originate at the neural crest during embryonic development [33], accounts for skin and hair pigmentation. It has been suggested that cytosolic proteins, such as the heat shock 70 kDa protein (HSP70) chaperone, can be recruited and sequestered in exosomes as a mere consequence of their physical interaction with other proteins [7]. The role of HSP70 in progressive depigmentation has been confirmed in vivo using a mouse model of autoimmune vitiligo [34]. Vitiligo is an autoimmune disorder that involves progressive depigmentation mediated by a T-cell response to melanocytes. The amount of HSP70 is increased in the supernatant of vitiligo versus the control melanocytes, and, when secreted into the extracellular space by melanocytes, HSP70 interacts with antigen-presenting dendritic cells, enhancing their uptake and processing of antigens. This leads to the activation of T-cells, which are ultimately responsible for the loss of HSP70-producing melanocytes. The mouse model used by Denman and colleagues was based on the introduction by gene gun vaccination of eukaryotic plasmids encoding melanocyte differentiation antigens. When combined with the vaccination protocol, the introduction of inducible HSP70 resulted in significantly accelerated depigmentation. In addition, this protein is found in exosomes derived from most cell types and it is therefore likely to occur in exosomes from melanocytes. In light of these observations, it is tempting to speculate that the release of HSP70-containing vesicles to the extracellular milieu by melanocytes may contribute to the disease.

Importantly, not only can the exosomes potentially secreted by melanocytes affect the pigmentation process, but also signaling molecules carried by exosomes originating in other cell types resident in the skin could affect melanocytes. In this regard, one sophisticated study has compared the effects of exosomes obtained from human keratinocytes of different skin phototypes to stimulate melanocyte function, as well as the potential of ultraviolet B (UVB) light to modulate this capacity. Interestingly, the expression of key proteins participating in the pigmentation process, such as the enzyme tyrosinase, the melanocyte isoform of microphthalmia-associated transcription factor (MITF), which is the master transcriptional regulator of melanogenesis, and the Rab27a protein, involved in the mobilization of melanosomes, were proven to be increased in melanocytes in the presence of exosomes either obtained from Caucasian donors and treated with UVB, or obtained from black donors [35]. This groundbreaking work demonstrated for the first time that miRNAs contained in exosomes secreted by keratinocytes have the ability to modulate pigmentation. On the other hand, another study has reported the ability of keratinocyte exosome-derived miR-675 to decrease MITF levels in melanocytes [36]. Altogether, these observations may prompt new strategies for modulating skin pigmentation and hair pigmentation through the potential effects driven by exosomes on HF bulge resident melanocyte precursors.

## 3. Role of Extracellular Vesicles in Hair Follicle Function

One previous study focused on rodent incisors found evidence of exosomes mediating epithelium-mesenchyme molecular crosstalk [37]. Since the HF is considered one of the prototypical systems for epithelial-mesenchymal crosstalk, it can be postulated as a top candidate to benefit from EV-based clinical approaches. Moreover, regenerative hair waves continuously occur over mice lifetimes and it has been demonstrated that the cycling rhythm declines with age, although it is possible to rescue the cycling capability by transplanting aged skin into a younger donor [38]. In addition, transplanted DP cells are able to induce hair growth [39,40]. In sum, these observations suggest that hair growth induction is rather dependent on secreted factors than a cell-autonomous process.

### 3.1. Effects of Extracellular Vesicles on Hair Follicle Dynamics

#### 3.1.1. Exosomes as Signaling Mediators with the Potential to Modulate Hair Cycling

Bone morphogenetic proteins (BMP), which emanate from dermal cells and adipocytes, maintain SC quiescence during telogen [3]. Interestingly, DP cells undergo oscillating and out of phase expression of BMP factors and of those inducing Wnt signaling, which allows for the fine regulation of HF dynamics [41] (Figure 1). Significant findings involving a link between skin and hair follicle regeneration and EVs have been compiled in Table 1, with emphasis on the signaling pathways that mediate these effects. It is well established that Wnt factors are master regulators of HF morphogenesis and hair growth [42]. In fact, epidermal Wnt ligands play a central role in wound-induced de novo hair formation in adult skin. Hence, they have been pointed out as potential targets for the treatment of hair-related syndromes like alopecia [43]. On the other hand, active Wnt factors have been identified as exosome-secreted molecules which can be contained in the interior compartment of these vesicles [44] as well as transported exteriorly [45]. Several studies have demonstrated that Wnt signaling in recipient cells can be mediated by horizontal transfer of the proteomic contents of EVs [46]. For instance, EVs from breast cancer cells have been shown to induce Wnt5a in macrophages, which in turn increases macrophage-induced invasiveness of the MCF-7 cell line [47]. In addition, Wnt signaling has been found to be activated in target cells by EVs containing both β-catenin, the major effector protein of the canonical Wnt pathway, and 14-3-3 proteins [48]. The latter can bind Dvl-2 and GSK-3β, which in turn mitigates the interaction between GSK-3β and β-catenin, therefore enhancing Wnt signaling. In fact, both Wnt4 and Wnt11 secreted in exosomes derived from human umbilical cord MSCs have been proven to benefit cutaneous regeneration in a rat skin burn model. Wnt4 has been found to enhance Wnt/β-catenin signaling and angiogenesis in the skin [25,49], while the treatment of MSCs with 3,3′-diindolylmethane has been shown to induce Wnt11 expression in exosomes in an autocrine fashion, also favoring wound healing through the activation of Wnt/β-catenin signaling [50]. It would be interesting to further investigate the strong effects of exosome-secreted Wnt11 on hair growth which have been suggested by the results of this study. In addition, fibroblast-derived exosomes appear to mobilize Wnt11-mediated autocrine signaling in breast cancer cells, promoting protrusive activity and motility through the Wnt-planar cell polarity signaling pathway [51]. Interestingly, differential subsets of Wnt-containing vesicles can be secreted in a distinctive manner in polarized epithelial cells, a mechanism that has been demonstrated for the release of Wnt3a and Wnt11 in MDCK cells [52] and which could be of interest in the well-organized architecture of the interfollicular epidermis and HF. In agreement with these observations, an upregulation in the expression of Wnt3a and Wnt5a, consistent with hair growth induction, has been found in mouse skin treated with intradermically-injected EVs obtained from MSCs [53]. Accordingly, exosomes obtained from human DP cells have been found to extend the anagen phase of the hair cycle by inducing the expression of β-catenin and Sonic hedgehog (Shh) when injected into mouse skin [54]. Interestingly, in addition to the important role that Shh signaling plays in hair morphogenesis, its homologous Hh has been shown to be present in exosome-like vesicles in *Drosophila* [55].

MicroRNAs (miRNAs) are small noncoding RNA molecules which are capable of altering gene expression post transcriptionally and are typically transported in EVs [56,57]. These molecules have been implicated in the control of skin and HF development through the modulation of Wnt signaling [58]. In a step forward, miR-181c contained in human umbilical cord MSC-exosomes was found to be a central player in attenuating burn-induced inflammation in a rat model [59]. Additionally, exosomes obtained from synovium-MSCs that overexpress miR-126-3p have been found to promote increased expression of P-AKT and ERK1/2 in HMEC-1 endothelial cells and contribute to skin wound healing in diabetic rats [27].

Several important signaling pathways involved in key cellular processes such as cell migration, proliferation, and survival are activated by epidermal growth factor (EGF) ligands binding their receptors on the plasma membrane. Among these, the routes involving Pi3K/AKT, MAPK/ERK, STAT3, and IGF1 have been connected with exosome-mediated effects on skin wound healing or hair growth [21,25,27,28,53]. For instance, the JAK/STAT pathway is implicated in hair growth [60]. Transforming Growth Factor (TGF)-α belongs to the EGF family and is upstream of the Grb2/Sos-Ras-Raf-MEK1,2-ERK1,2 signaling cascade, which is widely accepted as a promoter of cell proliferation [23]. Since mice with TGF-α deficiency display skin and hair abnormalities [61,62], TGF-α has been implicated in the control of HF shape [42]. TGF-α selectively stimulates hsp90α exosome-secretion in human keratinocytes, but not in dermal cells [23]. Interestingly, hsp90α promotes migration of dermal cells even in the presence of the strong inhibitor TGF-β, which is abundant in the skin wound environment. Thus, hsp90α exosome-secretion by keratinocytes in response to TGF-α may constitute a major factor stimulating cell motility in the wound bed [23]. In particular, since inhibitory signals mediated by TGF-β family factors are involved in the control of HF regression (catagen) in vivo [63,64], it would be interesting to investigate whether TGF-α stimuli can trigger exosome-mediated secretion of hsp90α by HF epithelial cells and affect hair cycle progression. More work is also needed to determine to what extent the induction in expression of the apoptosis suppressor BCL-2 in response to MSC-exosomes [53] could participate in the extension of the anagen phase.

#### 3.1.2. Use of Extracellular Vesicles to Stimulate Hair Growth: Evidence and Clues

Although only a few studies have focused on the use of exosomes to stimulate hair growth, the findings in this field are promising. A patented study has provided the first evidence of exosomes obtained from MSCs being a central component of a pharmaceutical composition directed at enhancing hair growth [65]. The induction of hair growth was accompanied by an improvement in wound healing, which is actually not surprising, since both processes have previously been linked by a number of studies, and different mechanisms involved in this link have been pointed out [66,67,68]. In agreement with these observations, subcutaneous administration of conditioned media from human amniotic fluid-derived MSCs in a full thickness wound model in rats has been found to promote hair regrowth, together with the acceleration of wound healing [69]. However, only a small number of studies with several limitations have so far been focused on the characterization of the mechanisms involved in the beneficial effects of exosomes on hair growth. Importantly, intradermically injected MSC-EVs have been shown to favor telogen to anagen transition in vivo in a mouse model [44]. An increase in the expression of proliferation, survival, and migration markers in DP cells treated in vitro with MSC-EVs was pointed out by the authors as a sign of stimulation, including induced expression of PCNA, P-AKT, P-ERK, and growth factors such as VEGF and IGF-1. However, since those traits are not typically found in DP cells over the hair cycle [65], determining the signaling molecules that mediate the induction of hair growth in response to treatment with EVs in vivo will be key to unequivocally unveiling the molecular mechanisms mediating this effect. In a recent study, human DP exosomes were injected into mouse skin to promote hair growth [54], which can mimic the physiological paracrine effects that DP cells exert on epithelial cells. An induction in β-catenin and Shh levels was detected in treated skin, as well as in epithelial hair follicle outer root sheath cells isolated from human scalps and cultured with DP exosomes. These results consistently revealed the participation of Wnt/β-catenin and Shh pathways in the molecular mechanisms driving hair growth in response to exosomes. Interestingly, anagen extension was due not only to a premature anagen onset but also to a delay of the catagen phase in mice. This suggests that additional molecular mechanisms responsible for hindering the transition to the catagen stage could be involved in enhanced hair growth. Finally, exosomes containing hTert have been proven to be secreted by cancer cells and incorporated by telomerase negative fibroblasts, turning them into nonmalignant cells with telomerase activity [70]. Since HF SC proliferation occurs as a consequence of conditional telomerase induction [71], hTert-containing exosomes could contribute to triggering the proliferation of slow-cycling SCs in the HF bulge and favor the transition from telogen to anagen, ultimately promoting hair growth.

Overall, these observations suggest that since EV cargo has the potential to target a wide range of molecular processes and recipient cells, EVs emerge as both natural mediators potentially participating in the control of the hair cycle and promising delivery vehicles for the improvement of skin and hair regeneration. More work needs to be done in order to determine both the physiological contribution of exosomes to the HF cycle in vivo and the therapeutic potential of the use of exosomes in the clinics in order to modulate hair growth.

### 3.2. Immune System Cells and Hair Follicles

**Figure 2:**
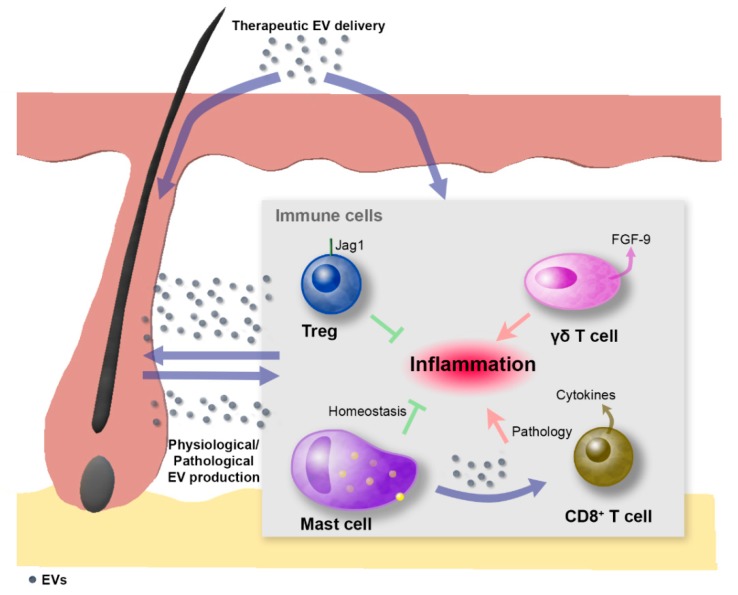
Extracellular-vesicle-mediated crosstalk between immune cells and hair follicles. EVs can be naturally produced under physiological/pathological conditions or, alternatively, can be therapeutically delivered. EVs can contribute to the modulation of hair follicle stem cell function by acting either directly or indirectly through their effect on immune cells. In this sense, different types of immune cells are involved in the control of hair follicle dynamics. Skin-resident regulatory T (Treg) cells that express high levels of the Notch ligand Jagged-1 (Jag1) facilitate hair follicle stem cell function and contribute to hair follicle regeneration. Skin resident mast cells contribute to hair follicle immune privilege under physiological conditions but are known to become proinflammatory in alopecia areata, in which mast cells contain less TGF-β1 and produce exosomes that induce T lymphocytes to proliferate and secrete cytokines. The γδ-T cell population in mouse skin secretes FGF-9, which modulates hair follicle neogenesis after skin wounding. In addition, epithelial and dermal hair follicle cells can secrete EVs that potentially target other hair follicle cell populations or skin resident immune cells, contributing to the modulation of local inflammation. Purple arrows indicate the flux of EVs; red arrows indicate proinflammatory stimuli; green T bars indicate anti-inflammatory stimuli.

Different types of EVs have been implicated by a myriad of studies in interactions that involve and affect immune cells [4,72]. Concurrently, important molecules that are released via EVs and participate in immunomodulation are also recognized as essential factors involved in wound repair [18]. For instance, TGF-β1 belongs to the secretome of mesenchymal stromal cells and is released via exosomes [73]. Additionally, the extracellular functions carried out by exosome-secreted hsp proteins include stimulation of immunological cytokine production, activation of antigen-presenting cells, and anticancer functions [74]. In light of this knowledge, immune cells are indeed key candidates which participate in the regulation of tissue and hair regeneration as exosome producer or recipient cells (Figure 2). Hence, therapeutic strategies for hair-related syndromes should also target immune cell populations. In this sense, several pieces of evidence indicate that important tissue regeneration processes are mediated by an intimate molecular dialog established between immune cells and other skin and HF resident cells. For instance, Fgf-9 secreted by γδ-T cells modulates HF neogenesis after skin wounding in adult mice through the activation of Wnt expression and Wnt signaling in skin fibroblasts [75]. In addition, regulatory T-cells have also been identified as promoters of proliferation and differentiation of HF SCs [76]. On the contrary, signals that inhibit hair growth have been conferred to molecules secreted by immune cells, such as prostaglandin D2 (PGD2), which blocks HF regeneration through the Gpr44 receptor. This opens up the possibility of therapeutically inhibiting PGD2 production or Gpr44 signaling to promote skin regeneration [77]. The opposed effects of different molecules transported via EVs are expected to be controlled depending on the status of the recipient cells (e.g., the surface receptors being expressed), as well as the tissue microenvironment. In the case of EVs potentially delivered with therapeutic purposes, the specific molecules loaded as cargo can be chosen.

The importance of the crosstalk between immune cells and skin and HF cells has also been highlighted by the classification of alopecia areata as an autoimmune disorder involving a switch of mast cells towards a proinflammatory phenotype [78]. Mast cells in alopecia areata display lower levels of TGF-β and secrete exosomes that mediate the induction of T-cells to proliferate and increase cytokine production (Figure 2). This could motivate the use of exosomes for the treatment of autoimmune diseases that affect hair loss, serving as delivery vehicles for signaling molecules that contribute to the immune privilege of HFs, for example by facilitating the suppression of major histocompatibility complex class I molecules or by inducing the expression of immune privilege guardians like TGF-β1/2.

In this context, the use of exosomes as carriers of danger-associated molecular patterns (DAMPs) and molecules that regulate T-cell function can exert either a direct effect on HF SCs or an indirect effect through the activation of immune cells involved in the inflammatory response. This can mimic molecular signals that participate in the activation of skin SCs in response to skin damage, although it remains unknown whether exosomes are involved in this type of mechanism during physiological skin homeostasis and repair. Strikingly, exosomes enriched in telomeric repeat-containing RNA (TERRA), which are non-coding RNAs that contribute to telomere function, have been identified [79]. These TERRA-enriched exosomes have been found to stimulate inflammatory cytokines from immune cells and thus have been postulated as telomere-specific alarmins. The authors have therefore proposed that TERRA molecules transported by these exosomes work as telomere-specific DAMPs which account for telomeric dysfunction and trigger an inflammatory response in the recipient cells. Given that the HF bulge SC niche is known to be enriched in cells with relatively longer telomeres [80], this mechanism could be relevant as a potential way to signal telomere attrition affecting the HF SC compartment, revealing stem dysfunction and aging, which are linked to hair-related syndromes. Another scenario where DAMPs could gain importance is in the involution phase of the hair cycle (catagen). This stage is characterized by synchronic keratinocyte apoptosis in the regressing proximal hair bulb and constitutes a unique model of dramatic but physiologically programmed epithelial cell death [81]. Since the release of apoptotic bodies by follicular epithelial cells has been reported [82,83,84] but their signaling capacities generally neglected, we propose that new insights into this process could help to understand the molecular mechanisms that orchestrate hair cycling.

## 4. Concluding Remarks and Future Directions

The emergent role of EVs in HF dynamics is likely to become a high-impact tool in cosmetic and skin regenerative biomedicine. In line with recent work revisiting the isolation and purification methods of different types of EVs [5,45,85], the unification of procedures used to obtain and administer EVs is needed in order to generate more reliable and comparable data, as well as to implement novel techniques for the in vivo characterization of EV-driven mechanisms related to HF biology. In this regard, witty approaches are needed to more deeply explore the physiological and pathological occurrence of EV crosstalk among different HF subpopulations. Good examples of such strategies are the two photon approach [86] and the combined intravital microscopy with genetic lineage tracing [87], which was conceived by Greco’s lab in an attempt to observe the release of exosomes by mutant cells within the upper portion of the HF and epidermis and their subsequent clearing by both epithelial and immune cells. In summary, the ease of accessibility of skin may be strongly advantaged with regard to both the possibility of implementing novel treatments and the potential to serve as a source of exosome-secreting cells.

## Figures and Tables

**Table 1:** The role of extracellular vesicles in signaling pathways with the potential to modulate hair cycling.

Signaling Pathway	Molecules Transported via EVs	Source of EVs	Highlights of the Study	Model Used to Test the Effects	Ref.
Canonical Wnt	β-catenin and 14-3-3 proteins	HEK293T, SW480	EV-mediated activation of Wnt signaling in recipient cells	In vitro: HEK293T, COS7, SW480	[48]
	Wnt4	HuUC-MSCs	HuUC-MSC exosomes facilitated wound re-epithelization and cell proliferation through the activation of Wnt signaling	In vitro: HaCaT, Ea.hy926, rat dermal fibroblasts In vivo: Rat skin 2nd degree burn injury	[25,49]
	Wnt11	HuUC-MSCs	Exosomal Wnt11 autocrine signaling in response to 3-3′-diindolylmethane increased markers of stemness in MSCs and favored wound healing	In vitro: HaCaT, rat dermal fibroblasts In vivo: Rat skin 2nd degree burn injury	[50]
	Wnt3a, Wnt11	MDCK, HEK293, fibroblast L cells	Different populations of exosomes carrying Wnt factors secreted by epithelial cells depending on the cell polarity and cell type		[52]
	Wnt3a, Wnt5a	Mouse BM-MSCs	EVs contributed to hair growth in mice by promoting telogen to anagen conversion of HFs	In vivo: Mouse skin	[53]
Wnt-planar cell polarity	Wnt11	Mouse fibroblast L cells	Mouse fibroblast-derived exosomes mobilized Wnt11-mediated autocrine signaling, promoting protrusive activity and motility	In vitro: MDA-MB-231 In vivo: SCID mice	[51]
Canonical Wnt; Shh	Not characterized	HuDPCs	Exosomes extended the anagen phase of the hair cycle in mice by inducing the expression of β-catenin and Shh	In vivo: Mouse skin	[54]
Hh	Hh	*Drosophila*	Hh transport via exosomes along cytonems	In vitro: Cl8	[55]
TLR4	miR-181c	HuUC-MSCs	Exosomes overexpressing miR-181c reduced burn inflammation by downregulating the TLR4 signaling pathway	In vivo: Rat full-thickness burn injury	[59]
EGF/EGFR	mi-126-3p	HuS-MSCs	Improvement in the healing capacity of wound dressings by incorporating exosomes derived from miR126-overexpressing HuS-MSCs, which led to the activation of AKT and ERK1/2 through phosphorylation	In vitro: Human dermal fibroblast, HMEC-1 In vivo: Full-thickness excisional skin wound in diabetic rats	[27]
	ERK1/2	BM-MSCs	Key pathways for wound healing including Akt, ERK, and STAT3, activated by MSC-exosomes	In vitro: Diabetic versus normal wound patient fibroblasts	[21]
	ERK1/2	HuEPCs	ERK1/2-mediated improved angiogenesis in response to exosomes with beneficial effects on wound healing	In vitro: HMEC-1 In vivo: Full-thickness excisional skin wound in diabetic rats	[28]
	TGF-α	HKCs	Stimulation of the secretion of hsp90α in exosomes by HuK-promoted migration of both epidermal and dermal cells	In vitro: Primary neonatal HKCs, dermal cells	[23]

The table compiles significant findings involving a link between skin and hair follicle regeneration and EVs, with emphasis on the pathways and the specific signaling molecules mediating these effects. Legend: BM-MSCs, bone marrow-derived mesenchymal stem cells; EGF, Epidermal Growth Factor; EGFR, Epidermal Growth Factor Receptor; EV, extracellular vesicles; Hh, Hedgehog; HKCs, human keratinocytes; HuDPCs, human dermal papilla cells; HuEPCs, human endothelial progenitor cells; HuS-MSCs, human synovium mesenchymal stem cells; HuUC-MSCs, human umbilical cord mesenchymal stem cells; Shh, Sonic hedgehog; TGF, Transforming Growth Factor.

## Abbreviations

BMP	Bone Morphogenetic Protein
DAMPS	Danger-Associated Molecular Patterns
DP	Dermal Papilla
EGF	Epidermal Growth Factor
ESCRT	Endosomal Sorting Complexes Required for Transport
EV	Extracellular Vesicles
HF	Hair Follicle
HSP70	Heat shock 70 kDa protein
miRNA	MicroRNA
MITF	Microphthalmia-associated transcription factor
MSC	Mesenchymal Stem Cell
MVB	Multivesicular Body
PGD2	Prostaglandin D2
SC	Stem Cell
Shh	Sonic Hedgehog
TERRA	Telomeric repeats-containing RNA
TGF	Transforming Growth Factor
UVB	Ultraviolet B
